# Managing patients with heart failure: contemporary real-world experience

**DOI:** 10.1186/s13104-022-05938-z

**Published:** 2022-02-10

**Authors:** Muhammad Siddiqui, Christopher Ripplinger, Hafsah Chalchal, Dakshina Murthy

**Affiliations:** 1 Department of Research, Saskatchewan Health Authority, Regina, SK Canada; 2 College of Medicine, University of Saskatchewan, Saskatoon, SK Canada; 3 Division of Cardiology, Regina General Hospital, Saskatchewan Health Authority, Regina, SK Canada

**Keywords:** Congestive HF, Management, Re-hospitalization

## Abstract

**Objective:**

Heart failure (HF) is a chronic disease with growing numbers of patients and a significant compromise in quality of life and high mortality. The main purpose of this study was to evaluate the current practices in managing patients with HF among patients admitted to the hospital and discharged with a primary diagnosis of HF and patients managed in the heart function clinic.

**Results:**

This study is a retrospective chart review of patients admitted to the hospital and discharged with a primary diagnosis of HF. A total of 448 patient charts were reviewed, of which 173 patients were in the hospital group and 275 patients in the Clinic group. 278 (62.1%) were men, and 170 (37.9%) were women. The Clinic group of patients were significantly received guideline-directed medical therapy (Beta-blockers, Angiotensin-converting enzyme inhibitors, Angiotensin receptor blockers, Diuretics, Mineralocorticoid receptor antagonists—p < 0.001). The Clinic group of patients (17.1%) were significantly less re-hospitalized (p < 0.001) compared to the Hospital group (28%) at 180 days. Physician led multidisciplinary Heart function clinics have better adherence to guideline directed medical therapy and significantly lower rates of re-hospitalization thereby providing cost effective heart failure management with usual care.

## Introduction

Heart failure (HF) is a major medical illness that affects both people and healthcare systems worldwide [1, 2]. HF is an emergent healthcare burden and one of the principal causes of hospitalizations and re-admission; it is expected to increase in prevalence over the next decade [2]. About 669,600 (3.6%) Canadian adults aged 40 years and older live with diagnosed HF, and approximately 92,900 (5.2 per 1000) Canadian adults aged 40 years and older received a new diagnosis of HF [3]. HF often does not occur in isolation and is often associated with other comorbidities, including hypertension, diabetes, coronary artery disease, and Valvular disease. Although various HF treatments have been developed, patients are often left with HF and discharged home diagnosis without clear instructions on what other lifestyle factors should be considered, such as diet, exercise, and smoking. This has led to the development of heart function clinics, which utilizes a multidisciplinary approach to HF by including cardiologists, dieticians, pharmacists, and nurses into the patients' circle of care.

Among heart HF patients, poor adherence to medications is a common problem. Inadequate compliance leads to increased HF exacerbations, reduced physical function, and a higher risk for hospital admission and death. Interventions to enhance medication compliance in HF patients have substantial effects on reducing re-admissions and decreasing mortality. Medication observance should be tackled in regular follow-up visits with HF patients, and interventions to improve compliance should be crucial for HF self-care programs [4].

To help out and manage symptoms, HF patients are taught self‐care strategies, maintain physical functioning, and prevent symptom exacerbations and deterioration of disease that could cause hospitalization or death. Medication is a critical part of HF management, and following medication regimes is a crucial behavior in HF self‐care. Unfortunately, HF patients' adherence to medication is low, which negatively affects clinical outcomes and leading to exacerbations of HF, reduces physical function, and causes a higher risk for hospital admission and death [5, 6].

Cardiovascular guideline-directed therapy has previously been shown to improve survival, reduce re-admissions to hospital, and improve life quality for those diagnosed with HF [2, 7]. Preventing re-admissions for HF patients is an increasing priority for clinicians, researchers, and various stakeholders. The purpose of this study was to retrospectively review patients being followed through the heart function clinic with a primary diagnosis of congestive HF. In doing so, we aim to analyze the current practice of diagnosis and management of congestive HF alongside re-admission and mortality rates while being followed up through the heart function clinic.

## Main text

### Methods

This study is a retrospective chart review of Heart Function Clinic, and patients admitted to the hospital and discharged with a primary diagnosis of HF from medicine department within an academic tertiary-care hospital system from Jan 2016 to July 2018. Only patients being recorded due to CHF's secondary cause, rather than congenital, were selected for this study. Patient data were retrieved from their respective charts and electronic medical records. Data on demographics, comorbidities, medication history, and re-hospitalization up to 180 days were obtained for both groups. Guideline directed medical therapy and re-hospitalization rates were the main focus of comparison between the two groups. Re-admissions to hospital and emergency room visits were detected on Sunrise Clinical Manager (SCM) electronic medical records and were separated based on the cause of re-admission.

One medical student reviewed charts of patients in the hospital group, and a second medical student reviewed charts of patients in the clinic group. The study cohort was identified using clinical data from the electronic health record. All data extracted from the patient records were recorded in a de-identified manner in a password-protected file and USB drive. Any information collected for the study was de-identified by assigning a unique study identification number to maintain confidentiality. All data was labeled with the participant ID, and no other identifying information was included in collecting data. Data were analyzed on a de-identified dataset to protect patient confidentiality.

For this study, it was impracticable to obtained informed consent because the participants may be deceased or have moved. Getting permission from family members might be problematic (e.g., due to change of address), not feasible because of a lack of funding and time, potentially disturbing to the family members. The research ethics board approved a waiver of informed consent as this study meets criteria a-f of Article 5.5 in the Tri-Council Policy Statement-2 (TCPS).

Statistical analysis was performed using SPSS Statistics software (Version 22.0. Armonk, NY: IBM Corp.). Data were expressed in frequencies, mean, and percentages. Chi-square test was used as a test of significance to compare differences between groups for categorical data, and t-test was used for continuous data. Significance was set at p < 0.05 level.

### Results

A total of 448 patient charts were reviewed, of which 173 patients were in the hospital group and 275 patients in the Clinic group. The mean age of included patients was 71.50 ± 13.9 years, and 170 (37.9%) were female. 255 (56.9%) of patients were older than 75 years of age. Our cohort's comorbid conditions were prevalent: 65.2% had hypertension, 33.5% had diabetes, 60.5% had hypercholesterolemia, and 52.2% had no history of smoking. Majority of study patients were with reduced ejection fraction (Table 1).

**Table 1:** Participants selected risk factors (n = 448)

	Total-448 n (%)	Hospital Group-173 n (%)	Clinic Group-275 n (%)	P-Value
Age				< 0.001
≤ 75 years	255 (56.9)	71 (41)	184 (66.9)	
> 75 years	193 (43.1)	102 (59)	91 (33.1)	
Gender				0.001
Coronary artery disease	217 (48.4)	80 (46.2)	137 (49.8)	0.46
Diabetes	150 (33.5)	67 (38.7)	83 (30.2)	0.06
Hypercholesterolemia	271 (60.5)	74 (42.8)	197 (71.6)	< 0.001
Hypertension	292 (65.2)	135 (78)	157 (57.1)	< 0.001
Peripheral vascular disease	27 (6)	18 (10.4)	9 (3.3)	0.02
Coronary artery bypass graft	98 (21.9)	37 (21.4)	61 (22.2)	0.84
Left ventricular hypertrophy (ECG)	52 (11.6)	22 (12.7)	30 (10.9)	0.56
Current smoker	70 (15.6)	25 (15.5)	45 (16.4)	< 0.001
Ejection fraction				< 0.001
Not done	57 (12.7)	57 (32.9)	0	
Normal	54 (12.1)	41 (23.7)	13 (4.7)	
Reduced < 50	337 (75.2)	75 (43.2)	262 (95.3)	
Angiography				< 0.001
No disease	70 (15.6)	12 (6.9)	58 (21.1)	
Single vessel disease	56 (12.5)	11 (6.4)	45 (16.4)	
Double vessel disease	34 (7.6)	11 (6.4)	23 (8.4)	
Tipple vessel disease	50 (11.2)	9 (5.2)	41 (14.9)	
Unknown	234 (52.2)	126 (72.8)	108 (39.3)	
Left main disease	4 (1.3)	4 (2.3)	0	

Most patients were prescribed ACE inhibitors (44.5% hospital gr; 65.8% clinic gr) or ARB (20.8% hospital gr; 20.7% clinic gr) before, and beta-blockers (76% hospital gr; 90.5% clinic gr) (Table 2). Furosemide was prescribed to 94.2% of a hospital patient and 66.9% of an HF clinic which evidently indicates that the two cohorts are different in disease stage and clinical stability. Almost two third (70.5%) of patients were not prescribed any mineralocorticoid; Aldactone was prescribed to 2.3% of hospital patients at discharge and 45.5% of heart clinic patients. In study population, only one heart function clinic patient had received Ivabradine and three patients received Angiotensin Receptor Neprilysin Inhibitor. The Clinic groups of patients were significantly on guideline-directed medical therapy (Beta-blockers, Angiotensin-converting enzyme inhibitors, Angiotensin receptor blockers, Diuretics, Mineralocorticoid receptor antagonists—p < 0.001).

**Table 2:** Participants medication of HF (n = 448)

	Total-448 n (%)	Hospital Group-173 n (%)	Clinic Group-275 n (%)	P-Value
Beta-blockers	381 (85)	132 (76.3)	249 (90.5)	< 0.001
Hydralazine	33 (7.4)	26 (15)	7 (2.5)	< 0.001
Nitrates	139 (31)	85 (49.1)	54 (19.6)	< 0.001
Diuretics	347 (77.5)	163 (94.2)	184 (66.9)	< 0.001
MRA				< 0.001
No	316 (70.5)	169 (97.7)	147 (53.5)	
Aldactone	129 (28.8)	4 (2.3)	125 (45.5)	
Eplerenone	3 (0.7)	0	3 (1.1)	
ARBs & ACE inhibitors				< 0.001
No	97 (21.6)	60 (34.7)	37 (13.5)	
ACE inhibitors	258 (57.6)	77 (44.5)	181 (65.8)	
ARBs	93 (20.8)	36 (20.8)	57 (20.7)	

**Fig. 1:**
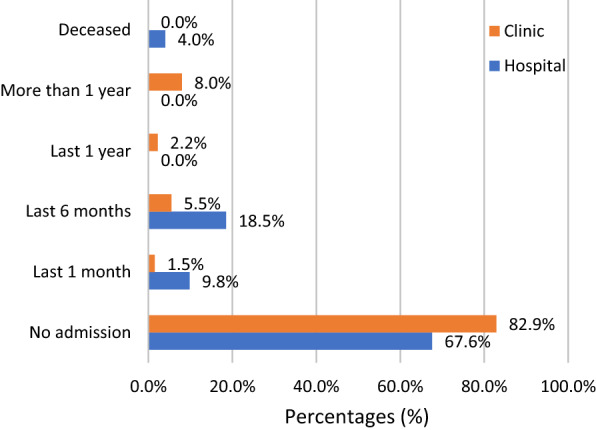
Patient's outcomes with a primary diagnosis of HF

Among 448 patients, concerning re-admissions to hospital, patients were less likely to be admitted after 1 month (1.5%) and 6 months (5.5%) of their initial visit to the heart function clinic (Fig. 1). Also, most patients were not re-admitted at any time following their initial assessment at the heart function clinic (82.9%) and among patients admitted to the hospital and discharged with a primary diagnosis of HF (67.6%). The Clinic group of patients (17.1%) were significantly less likely to be re-hospitalized (p < 0.001) compared to the Hospital group (28%) at 180 days.

### Discussion

This retrospective chart review study revealed interesting outcomes. Diagnosis and HF management are highly heterogeneous and complex and often yield mixed results in different HF populations [8–10]. Our center Heart Failure intervention program is leading by a Cardiologist with expertise in HF and cardiac specialist nurse. Additionally, Psychiatric interventions plus cardiac rehabilitation are also included in the program. Mainly, local and global left ventricle progressive dilatation, and dysfunction, due to secondary myocardial impairment in patients with heart failure and ischemic myocardiopathy [11]. In these patients, cardiac remodeling can lead to a decline in exercise ability and a rise in HF hospitalization [12].

Great interest has been shown by policymakers and researchers in the idea of averting re-admission rates among HF patients. The 30-day re-admission rate measures hospital performance; in the United States, it has been linked to financial penalties [22]. Moreover, at 6-month follow-up, re-admission within 30-day is coupled with a poor prognosis [23]. Results of a multicenter study conducted in 171 centers in which 43,143 patients were treated showed that the hospitals had a higher 1-year all-cause re-admission rate (54.7%) with high risk-adjusted 30-day re-admission rates (59.1%) [24]. In this study Clinic group of patients (17.1%) were significantly less likely to be re-hospitalized (p < 0.001) compared to the Hospital group (28%) at 180 days. A study conducted by Chen et al. [13] revealed that heart failure management programs had a lower 1-year re-admission rate (29.67%) than patients who dealt with regular treatment (38.5%). In other countries of HF registries, such as the HF outcome registry in India, the 1-year re-admission rate was 30.1% [14], and in the Saudi Arabia registry, it was 36% [15]. On the other hand, in patients hospitalized with acute HF in Europe, the EHFS-2 registry shows a 1-year mortality rate of 21.9% [16]. The HF registry indicates a 24.4% mortality rate in 1 year in India [14]. In the United Kingdom, a population-based cohort study conducted between 2000 and 2017 revealed that for people with a new HF diagnosis, the overall 1-year mortality decreased from 25.8 to 19.2% between 2000 and 2016 [17]. In comparison, our study's hospital group had a 1-year mortality rate of 4%, and the heart function clinic had 0%.

Mineralocorticoid receptor antagonist (MRA) therapy is one component of treating patients with systolic HF. Clinical trials have demonstrated that MRA therapy reduces morbidity and mortality in HF patients due to left ventricular systolic dysfunction. Our study results have revealed that MRA was underutilized (23%) in patients admitted to the hospital and discharged with HF's primary diagnosis. Overall, this study showed MRA's underutilization, which is consistent with prior studies, predominantly in acutely decompensated HF patients [18–20]. MRA's due to the risk of developing hyperkalemia and a combination of providers [20]. Other impediments consist of ambiguity about who should prescribe them in transitions of care, physician's expertise about patient suitability, apprehensions about adverse effects, polypharmacy, follow-up observance, and non-compliance [21, 22]. In patients with underlying chronic kidney disease, timely follow-up laboratory testing is critical in reducing hyperkalemia risk [23, 24]. Appropriate laboratory follow-up increases to 25.2% (inpatient MRA initiation) from 2.8% (outpatient MRA initiation) [25].

The aims of this study were focused on adverse outcomes. Therefore, we don't have a cost analysis for this study. We agree it's an essential issue for the heart function clinic program, and the results will provide helpful information for policy decision-makers. Comparisons with the hospital discharged group showed that the heart function clinic reduced recurrent events of the hospitalization. It is believed that the disease management program would be more cost-effective by decreasing the HF re-admission rate. Study results concluded that:

- Patients treated in the heart function clinic are treated accorded HF guidelines.
- All efforts should be made to guarantee that any patient admitted due to HF has an ultrasound done during the hospitalization or the first 2 weeks after discharge to classify them better and treat them according to guidelines.
- Protocols and pathways to refer patients to the HF clinic need to be implemented.

## Limitations

One of the core limitations of this study is that the patients studied were from a single tertiary care center. The study developed and evaluated the effectiveness of a heart function clinic managing HF patients over a 180 days follow-up period. Hence, the study results may not be generalizable to the spectrum of HF patients. Further investigation is warranted to assess the effectiveness of a specialized HF management program in a multicentre setup with a more extended follow-up period. Another limitation is the retrospective type of data. A number of situations may have been missed because the data was not transliterated or easily found in patients' charts. This study did not report cost or resource utilization for the cohort of patients. Thus, it cannot come to any conclusions regarding the unit's utility from a system perspective. Future research might explore what this means from a system perspective. We did not explore Sodium-glucose co-transporter 2 (SGLT2) inhibitors in this study, which have been shown to reduce heart failure hospitalizations in patients with heart failure with and without diabetes.

Physician specialist-led multidisciplinary heart function clinic managed HF patients better implement guideline-directed medical therapy and lower re-hospitalization rates than those treated by non-specialist Physicians. Comparisons with the hospital discharged group showed that the heart function clinic reduced recurrent events of the hospitalization.

## Data Availability

The datasets during and/or analyzed during the current study are available from the corresponding author on reasonable request.

## Abbreviations

HF: Heart failure
REB: Research ethics board
SCM: Sunrise Clinical Manager
TCPS: Tri-Council Policy Statement
MRA: Mineralocorticoid receptor antagonist
